# *Candida albicans* Shed Msb2 and Host Mucins Affect the Candidacidal Activity of Salivary Hst 5

**DOI:** 10.3390/pathogens4040752

**Published:** 2015-10-30

**Authors:** Sumant Puri, Justin Friedman, Darpan Saraswat, Rohitashw Kumar, Rui Li, Donna Ruszaj, Mira Edgerton

**Affiliations:** 1 Department of Oral Biology, School of Dental Medicine, University at Buffalo, Buffalo, NY 14214, USA; E-Mails: spuri@buffalo.edu (S.P.); jmfriedm@buffalo.edu (J.F.); darpansa@buffalo.edu (D.S.); rohitash@buffalo.edu (R.K.); rli7@buffalo.edu (R.L.); 2 Department of Pharmaceutical Sciences, University at Buffalo, Buffalo, NY 14214, USA; E-Mail: dmruszaj@buffalo.edu (D.R.)

**Keywords:** *Candida albicans*, oral candidiasis, Histatin 5, saliva, mucins, secreted aspartyl proteases

## Abstract

Salivary Histatin 5 (Hst 5) is an antimicrobial peptide that exhibits potent antifungal activity towards *Candida albicans*, the causative agent of oral candidiasis. However, it exhibits limited activity *in vivo*, largely due to inactivation by salivary components of both host and pathogen origin. Proteins secreted by *C. albicans* during infection such as secreted aspartyl proteases (Saps) and shed mucin Msb2 can reduce Hst 5 activity; and human salivary mucins, while suggested to protect Hst 5 from proteolytic degradation, can entrap peptides into mucin gels, thereby reducing bioavailability. We show here that Sap6 that is secreted during hyphal growth reduces Hst 5 activity, most likely a result of proteolytic degradation of Hst 5 since this effect is abrogated with heat inactivated Sap 6. We further show that just like *C. albicans* shedding Msb2, mammalian mucins, fetuin and porcine gut mucin (that is related to salivary mucins), also reduce Hst 5 activity. However, we identify mucin-like protein-induced changes in *C. albicans* cell morphology and aggregation patterns, suggesting that the effect of such proteins on Hst 5 cannot be interpreted independently of their effect on yeast cells.

## 1. Introduction

Histatin 5 (Hst 5) is the most potent antifungal peptide in saliva with candidacidal activity towards *Candida albicans* [1,2], the causative agent of oropharyngeal candidiasis (oral thrush). Although effective at its physiological concentration of 30 μM when tested *in vitro*, Hst 5 activity is compromised in the oral cavity by interaction with salts, metals, proteases, and other proteins in saliva [3,4]. Proteins that interact with Hsts in saliva can potentially be contributed by both the human host [5,6,7] as well as by the commensal and pathogenic microorganisms in the oral cavity, including *C. albicans* [8].

Secreted aspartyl proteinases (Saps) secreted by *C. albicans* during yeast and hyphal growth phase are important virulence factors that help in digestion of host components for nutritional purposes and in tissue invasion [9,10]. Hst 5 has been shown to be a potential substrate of Saps; Sap 9 in particular was shown to digest Hst 5 and thereby limit its candidacidal function *in vitro* [11]. Saps also have a potential role in cleavage of the *C. albicans* Msb2 head sensor protein of the Cek1 signaling pathway in response to environmental signals (such as *N*-acetylglucosamine and 37 °C), resulting in shedding of its extracellular domain [12]. Msb2 is a high molecular weight, heavily glycosylated mucin-like protein, and recent reports suggest that the glycosylated shed domain of Msb2 binds Hst 5 to negatively affect its antimicrobial activity [13]. Although binding of shed Msb2 to other antimicrobial peptides was shown to be dependent on its glycosylation status [14], Hst 5 binding to shed Msb2 has not been evaluated for the dependency on glycosylation. Interestingly, both Saps (particularly Sap 6) and Msb2 are major components of the *C. albicans* secretome [8].

Saliva contains a milieu of human secreted proteins [15,16] and potentially microbial proteins from oral colonizers. Salivary mucins MUC5B and MUC7 are among the most abundant proteins in salivary fluid [15] whose function is to coat and protect the oral mucosa. Mucins are responsible for providing lubrication over epithelial surfaces, in the gastrointestinal, urogenital, and respiratory tracts, as well as within the oral cavity [7]. Mucins can bind to various other proteins, both by protein–protein interactions as well as by interactions of their glycan moieties with other proteins [7]. Interaction of Hsts with MUC proteins has been documented; binding of Hsts 1, 3, and 5 to MUC5B was characterized by yeast two-hybrid systems [7]. Analysis of the residues on MUC5B involved in this binding suggested the role of protein-protein interactions rather than glycan-protein interactions. Similarly, MUC7 purified from bacterial protein expression systems, and therefore lacking *N*- and *O*-glycosylation, was shown to bind to Hst 1 [17], further suggesting that the interactions between Hsts and salivary mucins are primarily protein-protein interactions.

Since both salivary mucins and the mucin-like regions of *C. albicans* secreted Msb2 have been shown to bind Hst 5, we questioned whether these mucins might reduce Hst 5 candidacidal activity by complex formation to reduce the bioavailability of the much smaller Hst 5 peptide. Alternatively, mucins or sMsb2, as well as secreted Sap 6, might alter Hst 5 activity through changes in yeast cell metabolism upon mucin exposure, independent from direct mucin-Hst 5 interactions. Therefore, we examined the effect of shed domain of Msb2 as well as three related mammalian proteins (unglycosylated serum albumin, glycosylated fetuin, and heavily glycosylated gut mucin) on their ability to affect Hst 5 candidacidal activity. 

## 2. Results

### 2.1. Sap 6 Affects Hst 5 Candidacidal Activity

**Figure 1:**
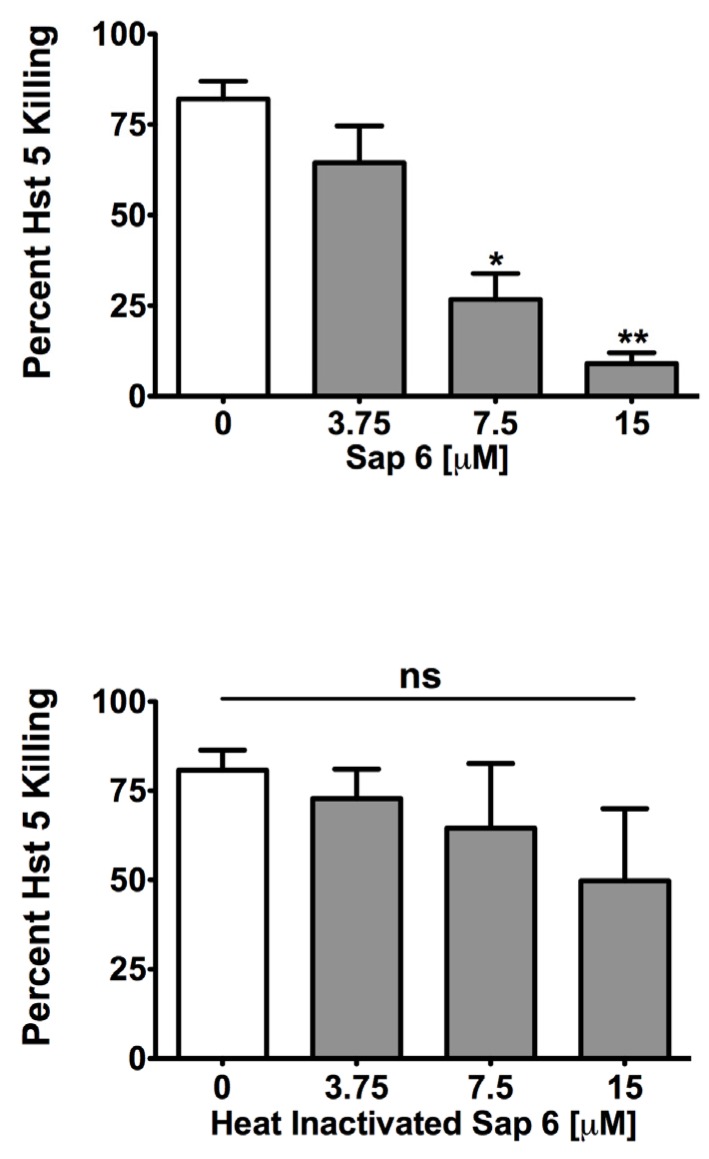
rSap 6 decreases Hst 5 candidacidal activity. (**Top**) Active rSap6 was added to 30 μM Hst 5 and incubated at 37 °C for 1 h, followed by candidacidal assay. Hst 5 activity showed a significant decrease in activity in the presence of rSap6, in a dose-dependent manner (**Bottom**) Heat inactivated rSap 6 had no significant effect on the activity of Hst 5. Statistical analysis was performed using 1 way ANOVA with Tukey Multiple Comparison test; significance at *p* < 0.05.

Secreted aspartyl proteases (Saps) can use Hst 5 as a substrate thereby affecting its activity by degradation [11]. We specifically tested the effect of Hst 5 interaction with Sap 6, one of the most abundantly secreted proteases during *C. albicans* hyphal growth phase [8], as well as during oropharyngeal candidiasis [9]. Hst 5 (30 μM) was incubated with rSap 6 (at 8:1, 4:1, and 2:1, Hst 5: protease) at 37 °C for 1 h and its candidacidal activity was then tested. At 4:1 and 2:1 ratios, rSap 6 caused significant reduction in Hst 5 activity, reducing killing to 26 percent and 9 percent, respectively, as compared to the 82% killing that was observed without protease (Figure 1, top). To substantiate whether this effect was dependent on the protease activity of Sap 6 and not related to its aggregation functions [18], we repeated the experiment with heat inactivated rSap 6. Heat inactivated rSap 6 did not affect Hst 5 activity (Figure 1, bottom), confirming that proteolytic activity of native Sap 6 is at least partially responsible for the reduction in the killing function of Hst 5.

**Figure 2:**
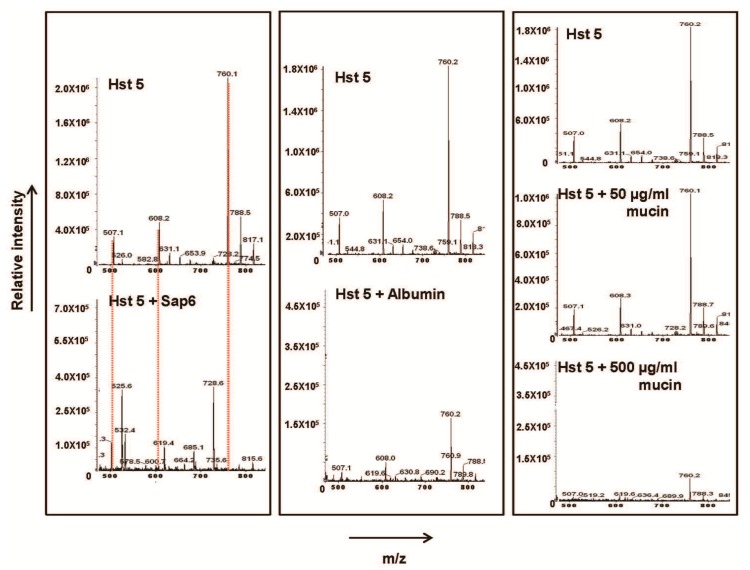
rSap 6 degrades Hst 5. Mass spectrometry of the reaction eluate of Hst 5 incubated with rSap6 (2:1,Hst5:rSap6), equimolar amounts of albumin, and 50 or 500 µg/mL porcine mucin, respectively, followed by spinning through a 10 Kd spin column showed that only Sap 6 degraded Hst 5. Degradation is observed here as the complete absence of all three major Hst 5 mass peaks: M + 4, 760 m/z; M + 3, 608; and M + 2507—marked here in red dotted line.

To further address whether Sap 6 degrades Hst 5, we spun the 2:1 Hst 5: Sap 6 incubation mixture through a Centricon^®^ 10 KDa molecular weight cut-off column that would allow Hst 5 and smaller peptides to pass through; and subjected the eluate to Liquid Chromatography/Mass Spectrometry. As seen in Figure 2, the M + 4 (760), M + 3 (608) and M + 2 (507) mass/charge (m/z) peak for intact Hst 5 completely disappeared in the reaction containing both Sap 6 and Hst 5, with generation of newer unique peaks with smaller m/z likely representing Hst 5 degradation products. Analysis of these fragments to determine Sap6-specific cleavage sites within Hst 5 is ongoing.

We further noted that the number of *C. albicans* control cells (without Hst 5 exposure) increased by twofold after incubation with rSap 6 only for 1 h, compared with those cells incubated with buffer alone. This increase in cell number was found following incubation with active as well as heat inactivated rSap 6. This suggested that the presence of Sap protein itself, independent of its protease function, affected *C. albicans* cell growth with respect to the conditions of our candidacidal assay. This was further confirmed by microscopic observations that showed both active and inactive rSap 6-treated *C. albicans* cells formed pseudohyphae and grew in longer chains, compared to untreated cells (data not shown).

### 2.2. Glycoproteins Affect Hst 5 Activity and *C. albicans* Growth Characteristics

**Figure 3:**
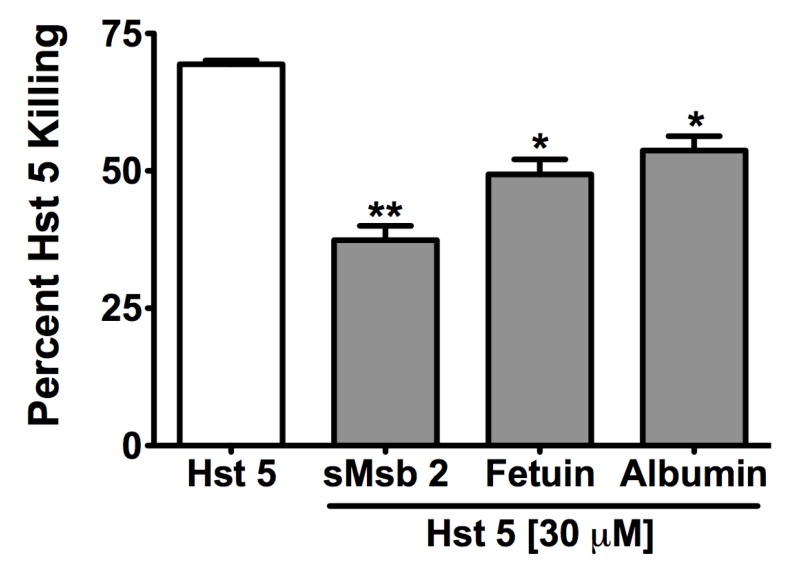
*C. albicans* and host mucins reduce Hst 5 activity. Equimolar purified shed domain of Msb2 and mammalian fetuin along with unglycosylated albumin control were incubated with 30 μM Hst 5 at 37 °C for 1 h, followed by candidacidal assay. All three proteins reduced Hst 5 activity; the most significant reduction was observed with sMsb2. Statistical analysis was performed using one-way ANOVA with Tukey Multiple Comparison test; significance at *p* < 0.05.

**Figure 4:**
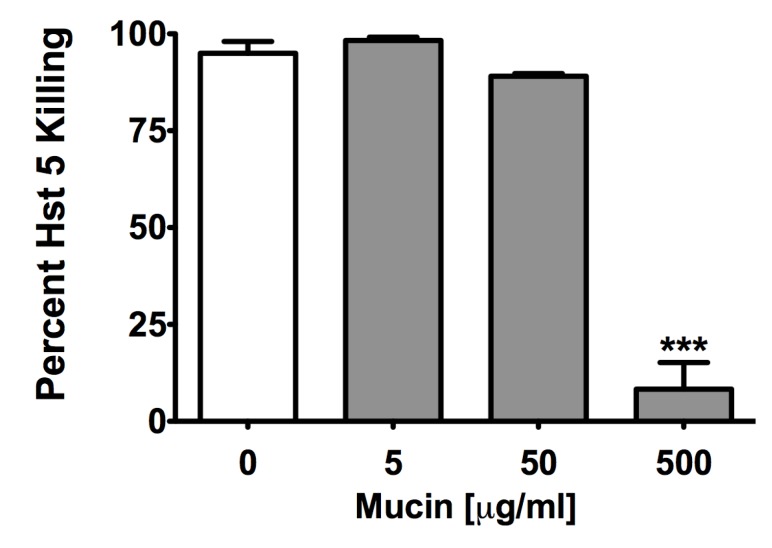
Porcine gut mucin decreases Hst 5 activity at higher concentrations. Different concentrations of porcine gut mucin were added to 30 μM Hst 5 and incubated at 37 °C for 1 h, followed by candidacidal assay. At the highest mucin concentration of 500 µg/mL, a significant decrease in percent killing was observed. Statistical analysis was performed using one-way ANOVA with Tukey Multiple Comparison test; significance at *p* < 0.05.

*C. albicans* Saps can also affect Hst 5 activity by their potential role in facilitating Msb2 shedding [12] since sMsb2 has been shown to bind Hst 5 to protect cells from its antifungal action [13]. Therefore, we examined whether this effect was specific to Msb2 or extends to other mucin-like or glycosylated proteins. Hst 5 (30 μM) was incubated with equimolar amounts of purified full-length shed domain of Msb2 (sMsb2), mammalian glycoprotein fetuin, and related unglycosylated albumin (as a control), respectively, and Hst 5 activity was assessed by a candidacidal assay. Both fetuin and albumin significantly reduced the candidacidal activity of Hst 5 after 60 min incubation from 70% to 50%, while purified sMsb2 caused the most significant reduction in Hst 5 activity to 38% (Figure 3). We further tested the effect of porcine gut mucin that is closely related to salivary mucins on Hst 5 activity. At lower mucin concentrations tested (5 and 50 μg/mL), there was no effect on Hst 5 activity. However, at a higher mucin concentration (500 μg/mL), there was a significant reduction in killing, from almost 95% to 8% (Figure 4). This was the largest reduction in Hst 5 activity as compared to all other proteins tested.

We next investigated whether the effect of these proteins on Hst 5 activity is related to complexation and Hst 5 sequestration or as a result, instead, of degradation caused by potential contaminating proteases that may be present in these commercial proteins. For this, 40 μM Hst 5 was incubated for 1 h at 37 °C with either 50 or 500 μg/mL porcine mucin or 40 μM albumin, respectively, and spun through a Centricon^®^ 10 KDa molecular weight cut-off column that would allow Hst 5 and smaller peptides to pass through followed by Liquid Chromatography/Mass Spectrometry analysis of the eluate.

As seen in Figure 2, incubation of albumin with Hst 5 caused a reduction in the peak intensity of all 3 m/z peaks for intact Hst 5 with no emergence of any significant lower m/z peaks that may represent degradation. Similarly, incubation with mucin led to a large dose-dependent decrease in the intensity of all three m/z peaks for Hst 5 (with greatest reduction in the presence of 500 μg/mL mucin) and no emergence of any significant lower m/z peaks. Incubation of Hst 5 with 500 μg/mL mucin resulted in almost complete disappearance of m/z peaks at 507 and 608, while the major Hst 5 peak (M + 4; 760) was still observed, albeit at lower intensity. Thus, unlike Sap 6, albumin or mucin did not cause the complete disappearance of Hst 5 from the reaction and did not generate any significant new mass peaks that may represent potential Hst 5 degradation products. This taken together with the fact that porcine mucin, which caused the highest reduction in Hst 5 activity, did not cause Hst 5 mass peaks to completely disappear (like Sap 6), strongly suggests that unlike Sap 6, these proteins do not affect Hst 5 activity as a result of degradation. However, all mass peaks outside of Hst 5 elution time frame were not evaluated; hence, a minor contribution of degradation caused by potential contaminating proteases could not be ruled out.

**Figure 5:**
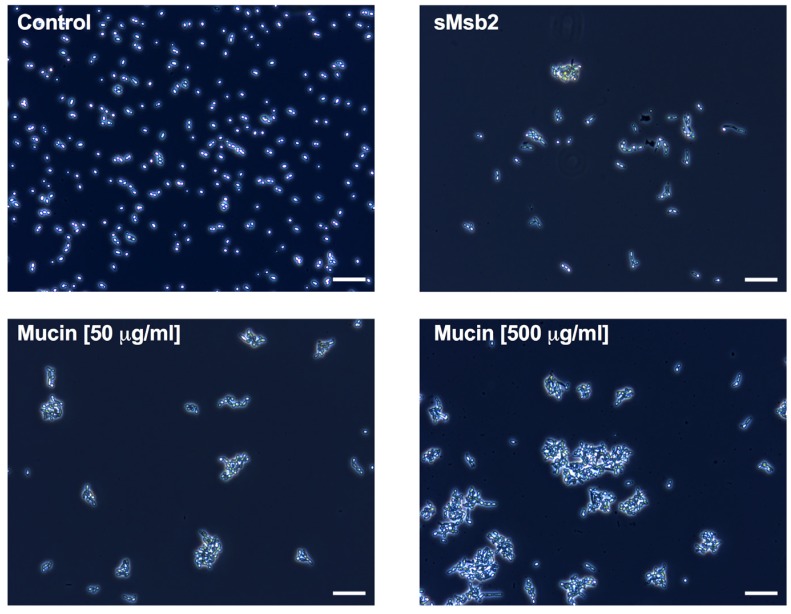
Mucins aggregate *C. albicans* cells. *C. albicans* cells were exposed to sMsb2 (13.7 µM) and porcine gut mucin (50 and 500 µg/mL) for 1 h and observed microscopically (phase contrast at 10X magnification). sMsb2 and gut mucin at both concentrations caused *C. albicans* cells to aggregate and induced pseudohyphal growth, as compared to untreated cells; gut mucin (500 µg/mL) induced large aggregates compared to sMsb2. Scale bar represents 50 µm.

Interestingly, when we assessed *C. albicans* cells microscopically that were treated with Hst 5 in the presence of sMsb2 and mucins, we observed unexpected changes in cell morphology and aggregation patterns (Figure 5). Both sMsb2 and porcine mucin induced pseudohyphal growth and cell–cell aggregation that was more pronounced for gut mucin, especially at higher concentrations. This mucin-induced aggregation of *C. albicans* cells altered our ability to count cells due to their clumping; thus, the apparent reduction in candidacidal activity of Hst 5 with mucin is an artifact and largely due to cell clumping reducing the apparent CFU. However, the effect of sMsb2 on Hst 5 activity could also be an indirect result of its influence on C. *albicans* Cek1 signaling and resulting cell surface changes. Since the high concentrations of purified sMsb2 used in these assays may not be relevant *in vivo*, we next examined the susceptibility to Hst 5 by *C. albicans* cells that have truncated versions of the shed domain of Msb2, varying also in the degree of shedding (19).

### 2.3. Msb2 Shed by *C. albicans* Does Not Uniformly Protect Cells against Hst 5

**Figure 6:**
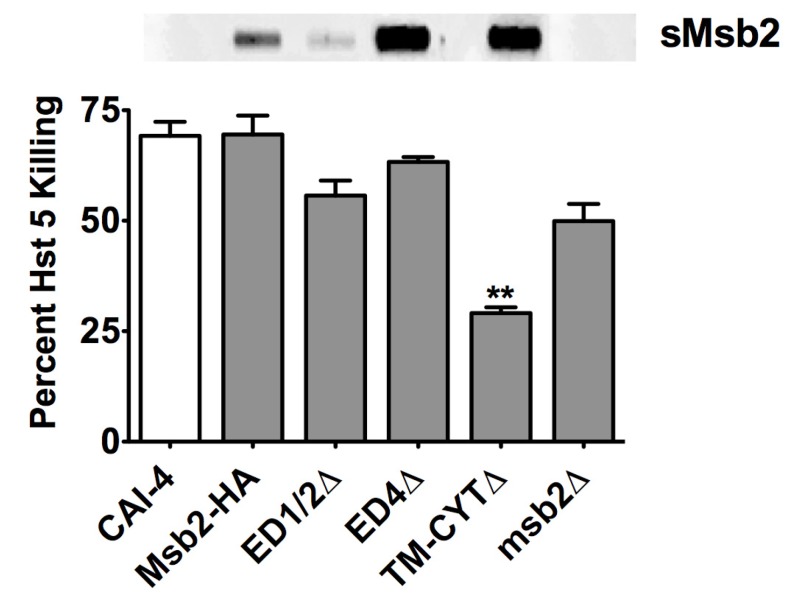
Msb2 shed by *C. albicans* does not always protect cells from Hst 5. Hst 5 susceptibility of various *C. albicans* Msb2 domain mutant cells was tested and compared with the levels of Msb2 shedding (shown in slot blot above). All strains have Hst 5 killing similar to WT except *TM-CYT*Δ that showed significant reduction in Hst 5 activity. *msb2*Δ cells with no shedding only showed a slight reduction in killing. CAI-4 cells with untagged WT Msb2 were used as the control. Statistical analysis was performed using student *t*-test; significance at *p* < 0.05.

To test whether Msb2 shed by *C. albicans* can protect cells from Hst 5, we performed candidacidal assays with various HA-tagged Msb2 domain mutant cells that varied in the size and level of Msb2 shedding (as detected by slot blotting for the HA-tagged shed domain in the culture medium) [19]. Cells (*ED1*/*2*Δ) lacking the *N*-terminal 100–900 residues that release a truncated Msb2 shed domain; cells (*ED4*Δ) lacking residues in the cleavage domain of Msb2 that constitutively shed Msb2 due to loss of cell tethering as well as cells (TM-CYTΔ) lacking the transmembrane and cytoplasmic signaling domain of Msb2 were used [19]. Both *ED1*/*2*Δ and *ED4*Δ cells showed killing similar to WT Msb2-HA cells (*p* > 0.05) (Figure 6), although the *ED1*/*2*Δ cells released a much smaller sized Msb2 (that would be expected to have less effect [19]) while *ED4*Δ cells released much higher levels of full length Msb2 (that would be expected to have more inhibition of Hst 5 as compared to WT [19]) (Figure 6 top panel). In contrast, *TM-CYT*Δ cells that released intact shed domain of Msb2 at levels similar to that of *ED4*Δ cells (Figure 6 top panel) showed reduced killing (Figure 6), most likely a result of lack of Cek1 signaling in the absence of the cytoplasmic domain, as previously shown [19]. Furthermore, killing was not significantly reduced in cells lacking Msb2 (and thus no shedding), when compared to other sMsb2 defective strains (Figure 6). Thus, Msb2 levels shed by *C. albicans* cells *in vivo* do not always afford protection against Hst 5, as compared to that seen with the addition of exogenously added purified sMsb2 *in vitro* (Figure 3).

## 3. Discussion

Mucin barriers have been shown to concentrate negatively charged peptides *in vitro* [20]. Hst 5 is a small cationic peptide with a globular configuration in aqueous environments [21], making epitope based protein-protein interactions unlikely. However, the net positive charge allows specific interactions with negatively charged fungal cell envelope [21]. We show here that mucin-like glycoproteins can affect Hst 5 activity. This suggests the possibility that mucin-like proteins might bind Hst 5 based on its charge in a non-discriminate manner, as seen here with the promiscuity of Hst 5 interactions with fetuin, albumin, porcine mucin, and sMsb2. Thus, Hst 5 activity can be modified by glycosylated proteins, and perhaps by other salivary glycoproteins such as the most abundant salivary protein, amylase [15].

Salivary mucins are most likely available in excess as compared to Hst 5 as Muc proteins are some of the most plentiful salivary proteins [15]. It has been suggested that mucins interact with Hst 5 to protect it from proteolytic degradation or function as a carrier to distribute it to other sites within the oral cavity, thereby potentially enhancing its activity [7]. However, our work shows that this interaction, at least *in vitro*, is detrimental for Hst 5 activity. It remains to be determined whether these opposing effects are a function of Hst 5 conformation that can change from globular in aqueous environments to α-helical in membrane mimetic environments [21]. Furthermore, Hst 5 entrapment by mucins may allow for its concentration in a mucin gel barrier that allows for delivery to mucosal surfaces, the site for *C. albicans* growth in the oral cavity. Hst 5 can then be potentially liberated from the mucin gel at these sites by the action of salivary enzymes.

While salivary proteome studies may reflect *in vivo* protein concentrations for the oral cavity, *C. albicans* secretome studies determine proteins in the supernatant of cells grown in liquid cultures, under shaking conditions, *in vitro*. Thus, it is not clear whether levels of Msb2 shed by *C. albicans in vivo* are constantly available in concentrations as high as previously tested *in vitro* to provide protection to *C. albicans* cells against Hst 5. On the other hand, enzymes can function at concentrations that are much lower than that of their substrates. This makes the effect of Saps on Hst 5 activity of greater relevance than shed Msb2. Although a previous report has shown the effect of Sap 9 on Hst 5 activity [11], Sap 9 and Sap 10, unlike other Saps, are Glycophosphatidylinositol (GPI)-anchored, thereby limiting their secretion. We believe that the effect of Sap 6 shown in this study may play a larger role in Hst 5 activity.

Aggregating pathogenic cells to enhance their clearance with salivary flow is one of the presumed roles of salivary mucins in preventing oral diseases (reviewed in [22]). This report, along with previous studies [23], confirms that mammalian mucins can affect *C. albicans* growth characteristics. We also observed changes in growth pattern and morphology with Sap 6 that were independent of its proteolytic function, ruling out nutritional advantage leading to greater cell count. It seems that Sap 6 may influence cell growth by signaling mechanisms. It is thus important to note that the interaction of Hst 5, with proteins such as mucins or Saps that affect *C. albicans* growth, aggregation, and morphology, may complicate the very interpretation of Hst 5 killing ability. This is because a crucial aspect of such assays is the cell shape, morphology and cell surface characteristics. Cellular aggregation, such as that seen in Figure 5, can lead to reduced Hst 5 accessibility to individual cells, creating an artificial reduction in killing or an apparent reduction in CFU count. Therefore, analyzing the effect on Hst 5 activity of host and *C. albicans* proteins affecting *C. albicans* growth characteristics requires cautious interpretation.

## 4. Materials and Methods

Strains, media, and proteins—*C. albicans* CAI-4 was cultured in yeast extract peptone dextrose (YPD) medium with uridine (50 μg/mL).

*C. albicans* strains with HA-tagged Msb2 and its domain mutants were constructed previously [12,19]. These mutants consisted of versions of Msb2 lacking each of the major functional domains: *ED1*/*2*Δ lacking residues 100–900 of the glycosylated extracellular region so that shed Msb2 is truncated with low glycosylation; *ED4*Δ cells lacking Msb2 residues 900–1250 within the cleavage domain of Msb2 resulting in constitutive shedding of Msb2 due to loss of cell tethering; and TM-CYTΔ cells lacking residues 1250–1409 that comprise the transmembrane and cytoplasmic signaling domain of Msb2 so that Msb2 is cleaved and shed in the absence of signaling. Briefly, Msb2 deletion derivative strains were constructed by a PCR-based approach [24] using the URA-Blaster technique [25]. PCR primers were designed to amplify the 3HA-URA3–3HA cassette in plasmid pCaMPY-3XHA [26], tailed with an additional 80 nucleotides of sequence flanking the open reading frame (ORF) of the region to be disrupted. PCR products were verified by gel electrophoresis. The purified PCR product (10 μg) was transformed into the Δmsb2/MSB2 strain with Frozen-EZ Yeast Transformation II Kit (Zymo Research, CA, USA). Uracil-deficient agar (YNB-URA) media was used for selection of URA positive colonies. PCR-based analysis of transformants was performed with primer pairs internal to the wild-type locus and the URA cassette. Homozygous transformants were reverted to the URA negative phenotype by selection on 5-fluororotic acid (5-FOA). Domain deletion knockouts were verified by immunoblot analysis.

Recombinant Sap6 (rSap6) was purified as described previously [18]. Shed domain of HA-tagged Msb2 was purified from the supernatant of CAI-4 cells expressing HA-tagged Msb2. Briefly, cells from an overnight culture were regrown in 500 mL and supernatant was collected, filtered through a 0.22 μm filter, and concentrated to 10 mL using a 100 kd cut-off Viva cell 100 column and buffer exchanged with Tris-buffer saline. HA-Msb2 was purified from this concentrated, buffer-exchanged supernatant using a pre-equilibrated anti-HA column. Albumin (A2153), fetuin (F2379), and porcine gut mucin (M1778) were purchased from Sigma Aldrich.

Candidacidal assays—The susceptibility of *C. albicans* cells to Hst 5 was measured using microdilution plate assays in triplicate as previously described [27], with some modifications for experiments involving rSap6 and mucins. Briefly, Hst 5 was incubated with the required concentration of respective proteins for 1 h at 37 °C before performing the candidacidal assay. For heat inactivated control of rSaps, protein was heat inactivated at 120 °C for 10 min before using in the candidacidal assay.

Proteolytic stability—For Hst 5 degradation analysis, Hst 5 was incubated with the required concentration of respective proteins for 1 h at 37 °C; and the reaction mixture was spun through a Centricon^®^ 10 KDa cut-off column (Millipore UFC501024, Amicon Ultra-0.5 centrifugal filter unit with Ultracel-10 membrane), diluted in water with 2%TFA (trifluoroacetic acid) to 40 ug/mL of peptide, and analyzed by Liquid Chromatography/Mass Spectrometry utilizing a C18 column and an API3000 triple quadrupole mass spectrometer (Applied Biosystems, Foster City, CA,USA). The mass spectrum of Hst 5 gave a distribution of charge states with the most abundant being the M + 4 at 760 Da.

Microscopy—Microscopic analysis was performed to qualitatively view the effect of sMsb2 and porcine gut mucin on the cells. Cells were grown overnight at 30 °C and cells were diluted in the morning to OD_600_ of 0.3 and allowed to grow for another 3 h until an OD_600_ of 0.8–1.0 was attained. The cells were then added to various tubes containing 13.7 μM sMsb2 or 50 μg/mL and 500 μg/mL porcine gut mucin, respectively, for 1 h at 30 °C. Phase contrast microscopy was performed using Zeiss Axio Scope.A1 microscope to compare cells exposed to various proteins with untreated cells. Images were taken at 10x magnification.

Slot blotting—HA-tagged Msb2 was detected by slot blotting *C. albicans* cells supernatant as described previously [12].

## 5. Conclusions

Hst 5 is susceptible to degradation-mediated inactivation and binding-mediated sequestration, both reducing its candidacidal activity in saliva *in vivo*. Here we showed that *C. albicans* Sap6 degrades Hst 5; while both *C. albicans* shed mucin Msb2 and host mucins bind Hst 5 thereby reducing its effective bioavailability. However, Hst 5 mediated killing is also modulated by fungal cell-cell aggregation as well as changes in fungal cell morphology. Thus, Hst 5 activity is changed by its sequestration with mucin-like proteins of fungal and host origin as well as its degradation by fungal proteinases.
